# Integrated Metabolomics and Transcriptomic Analysis of Hepatopancreas in Different Living Status *Macrobrachium nipponense* in Response to Hypoxia

**DOI:** 10.3390/antiox11010036

**Published:** 2021-12-24

**Authors:** Lei Xu, Wenyi Zhang, Hui Qiao, Sufei Jiang, Yiwei Xiong, Shubo Jin, Yongsheng Gong, Hongtuo Fu

**Affiliations:** 1 Wuxi Fisheries College, Nanjing Agricultural University, Wuxi 214081, China; xwmr258@163.com; 2 Key Laboratory of Freshwater Fisheries and Germplasm Resources Utilization, Freshwater Fisheries Research Center, Ministry of Agriculture, Chinese Academy of Fishery Sciences, Wuxi 214081, China; zhangwy@ffrc.cn (W.Z.); qiaoh@ffrc.cn (H.Q.); jiangsf@ffrc.cn (S.J.); xiongyw@ffrc.cn (Y.X.); jinsb@ffrc.cn (S.J.); gongys@ffrc.cn (Y.G.)

**Keywords:** hypoxia, metabolism, transcriptome, *Macrobrachium nipponense*, *PEPCK*

## Abstract

As the basic element of aerobic animal life, oxygen participates in most physiological activities of animals. Hypoxia stress is often the subject of aquatic animal research. *Macrobrachium nipponense*, an economically important aquatic animal in southern China, has been affected by hypoxia for many years and this has resulted in a large amount of economic loss due to its sensitivity to hypoxia; Metabolism and transcriptome data were combined in the analysis of the hepatopancreas of *M. nipponense* in different physiological states under hypoxia; A total of 108, 86, and 48 differentially expressed metabolites (DEMs) were found in three different comparisons (survived, moribund, and dead shrimps), respectively. Thirty-two common DEMs were found by comparing the different physiological states of *M. nipponense* with the control group in response to hypoxia. Twelve hypoxia-related genes were identified by screening and analyzing common DEMs. GTP phosphoenolpyruvate carboxykinase (*PEPCK*) was the only differentially expressed gene that ranked highly in transcriptome analysis combined with metabolome analysis. *PEPCK* ranked highly both in transcriptome analysis and in combination with metabolism analysis; therefore, it was considered to have an important role in hypoxic response. This manuscript fills the one-sidedness of the gap in hypoxia transcriptome analysis and reversely deduces several new genes related to hypoxia from metabolites. This study contributes to the clarification of the molecular process associated with *M. nipponense* under hypoxic stress.

## 1. Introduction

Oxygen is an essential environmental factor participating in the entire life of aquatic animals, including feeding, growth, and reproduction. Aquatic animals are prevented from being widely distributed at high altitudes and in underground regions due to oxygen demand. Since the discovery of the first hypoxia-related gene, erythropoietin (*EPO*), a series of genes related to hypoxia response mechanisms have been identified [1,2]. The identification of these genes has promoted the study of the hypoxia metabolic pathway in aquatic animals to a certain extent. However, a theoretical system of hypoxia has not formed. Until the discovery of the hypoxia-inducible factor (*HIF*), the basic molecular mechanisms of the response to hypoxia had not been determined [3,4,5,6]. With further research on *HIF*, a similar hypoxia metabolic pathway was found between aquatic animals and mammals. Thereafter, the discovery of hypoxia-related genes in aquatic animals entered a new stage, and a lot of hypoxia-related genes in mammals were cloned homologously into aquatic animals. At this stage, some important hypoxia-related genes were identified, including a series of well-known antioxidant genes [7,8,9].

However, as research continued, there were fewer and fewer homologous-cloned genes to be discovered, and the molecular mechanism of hypoxia was still not fully understood. When the research reached an impasse, the maturity of high-throughput DNA sequencing techniques saved the situation in time [10]. The study of hypoxia-related genes began to enter the transcriptome analysis era and this has resulted in a huge amount of available information, including the accuracy of proving that previous homologous-cloned genes are indeed related to hypoxia and a large number of unknown hypoxia related genes [11,12,13,14]. However, such huge amounts of data not only bring with them the hope of revealing the molecular mechanism of hypoxia, but they also increase the difficulty of screening useful genes. To verify the accuracy of the transcriptome and to find hypoxia-related genes quickly, the top differentially expressed genes (DEGs) are usually studied first. This method of artificially selecting DEGs will ignore a large number of DEGs that may play a crucial role in the response to hypoxia. Therefore, to improve screening the results of high-throughput sequencing, the joint analysis of metabolic and transcriptomic analysis has gradually entered the public field of vision. This method has been widely used in animals by searching for differential metabolites and DEGs, respectively, and then jointly analyzing them to find common differential genes to lock in genes that are related to the purpose of the research [15,16,17,18].

*Macrobrachium nipponense* (de Haan, 1849) is a popular and economically important aquatic shrimp in southern China due to its delicious taste and nutritional value. According to previous studies, the lethal concentration (LC50) of oxygen in *M. nipponense* is higher than it is in *Metapenaeus ensis* [19]. The experience of production also confirms that *M. nipponense* is less tolerant to hypoxia than other crustaceans. Therefore, it is of great importance to understand the molecular mechanism of hypoxia in *M. nipponense*. Although some studies have investigated the transcriptome of *M. nipponense* in response to hypoxia, these studies only have a control group and a survival group [20,21]. The limitation of this study is in identifying genes that are involved in the initial adaptation to hypoxia. In a previous study we have recognized these limitations and set up three comparisons between the control group and different states of survival [13]. Several of DEGs were thus identified in the previous study. To further establish whether the DEGs were closely related to hypoxia in previous transcriptomic analysis, a method of combining the analysis of metabolome and transcriptome data is used to further screen the transcriptome results.

To analyze our knowledge, a combined analysis of metabolome and transcriptome data associated with *M. nipponense* in different physiological states under hypoxia has not been reported previously. This study is devoted to identifying the essential genes that play a key role in response to hypoxia and provide the basic information to improve hypoxic resistance. This manuscript will assist in clarifying the molecular mechanisms used by *M. nipponense* in response to hypoxia. 

## 2. Materials and Methods

### 2.1. Sample Preparation and Gather 

In total, 100 healthy *M.nipponense* of about 2.0 ± 0.5 g were obtained from the breeding farm belonging to the Chinese Academy of Fisheries Science. To reduce the influence of transfer and make sure all the shrimps could adapt to the new environment before the hypoxic experiment, the shrimps were bred like those on the farm (18–30 °C, pH ≤ 9). After two weeks, the shrimps were randomly divided into a control group (6.0 ± 0.5 mg⋅L^−1^) and hypoxia group (1.0 ± 0.2 mg⋅L^−1^). The oxygen concentration in the hypoxia group was maintained with N_2_ gas, as previously described [8]. Thirty hepatopancreases were gathered from control, survived, moribund, and dead shrimps after hypoxia for eight h [13]. All the samples were immediately frozen in liquid nitrogen prior to storage at −80 °C.

### 2.2. Metabolites Extraction

In line with previous studies, the sample was extracted with 0.24 mL of extraction solution, and 10 μL of adonito was added; the sample was then vortexed, homogenized, and subjected to ultrasonication. After centrifugation, 25 μL was transferred to a clean 2 mL GC/MS glass bottle and collected as quality control (QC) samples. The samples were dried completely in a vacuum concentrator without heating. Then 30 μL of Methoxy amination hydrochloride was added and incubated at a constant temperature (70 °C) for 1.5 h. FAMEs (5 μL) was added to the QC sample while it was cooling to room temperature. All samples were analyzed by a gas chromatograph system coupled with a Pegasus HT time-of-flight mass spectrometer (GC-TOF-MS) [22].

### 2.3. GC-TOF-MS Analysis

GC-TOF-MS analysis was performed using an Agilent 7890 gas chromatograph system coupled with a Pegasus HT time-of-flight mass spectrometer. A corresponding DB-5MS capillary column was used according to the needs of the system. 1 μL of sample was injected in splitless mode and helium was used as the carrier gas. The energy in electron collision mode was −70 ev. After a solvent delay of 6–12 min, mass spectrometry data were obtained in a full scan mode in the *m*/*z* range of 50–500 at a rate of 20 spectra per sec. All relevant parameters were set according to previous studies [23].

### 2.4. Data Preprocessing and Annotation

Chroma TOF 4.3X software of the LECO Corporation and LECO-Fiehn Rtx5 database was used to extract, process, and normalize the original data. Both the mass spectrum match and retention index match were considered in metabolites identification. Peaks detected in <50% of QC samples or RSD > 30% in QC samples were removed.

### 2.5. Data Validation 

#### 2.5.1. RNA-Seq by qRT-PCR

Combined with the previous transcriptome database (PRJNA656359) [14], 12 differential genes (DEGs) were identified based on the common differential metabolites in the three comparative metabolome libraries (control vs. survived, control vs. moribund, control vs. dead). These DEGs were selected to validate the metabolism results. The tissues used for qRT-PCR were the same as those used in the metabolome. In subsequent experiments, the extraction of total RNA, the synthesis of cDNA, and the designs of qRT-PCR primer were completed as described in previous reports [14]. The primers for qRT-PCR are listed in Table S1. In line with previous studies, eukaryotic translation initiation factor 5A (EIF) was selected as the reference gene for qRT-PCR [24]. Three replications were used for each sample. The 2^–ΔΔCT^ method was chosen to calculate the expression of the selected genes.

#### 2.5.2. Identification of Key Metabolites in *M. nipponense*

Two hypoxia experiments were designed to verify the key metabolites. Firstly, 240 healthy adult *M. nipponense* were obtained and acclimated, as described above. Four groups were set with different oxygen conditions (1.5 ± 0.2 mg O_2_ L^−1^, 3.0 ± 0.2 mg O_2_ L^−1^, 4.5 ± 0.2 mg O_2_ L^−1^, and 6.0 ± 0.2 mg O_2_ L^−1^). After acclimating for two weeks, all the shrimp were randomly divided into four groups. Each group had three biological replications. The oxygen conditions were maintained by the method described before. After 8 h of hypoxia, six hepatopancreas were collected from the surviving prawns in each group. The samples were immediately frozen in liquid nitrogen before storage at −80 °C.

The second experiment was designed to verify the expression of key genes at different time points after hypoxia. About 300 healthy adult *M. nipponense* (2.0 ± 0.5 g) were prepared, as described previously. These shrimps were randomly divided into normal groups (6.0 ± 0.2 mg O_2_ L^−1^) and hypoxia groups (2.0 ± 0.2 mg O_2_ L^−1^). Each group had three biological repeats. As described previously, the oxygen conditions were kept constant. The target tissues were collected at 0, 6, 12, and 24 h during the hypoxia process. The method of collecting samples was as described above.

The qRT-PCR verification method of screened hypoxia-related genes in these two experiments was as described above. 

### 2.6. Statistical Analysis 

All confirmatory experiments had biological duplication (n = 3, n = 6 in metabolism). The appropriate method, including ANOVA and paired sample *t*-test of SPSS 20.0, were selected to analyze the variance of all data with *p* ≤ 0.05 considered to be significant [25].

## 3. Results

### 3.1. Principal Component Analysis (PCA) Analysis of Metabolism Profile 

**Figure 1:**
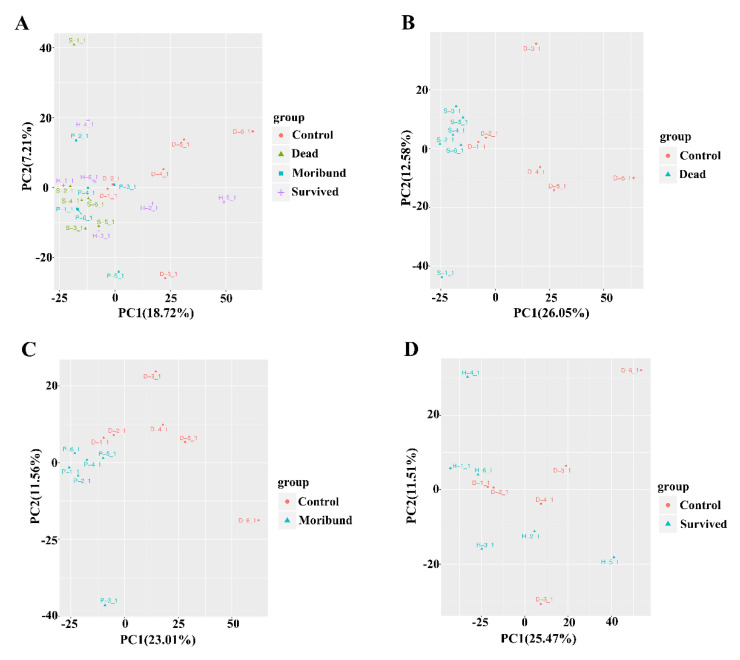
PCA analysis of all the samples (**A**). PCA analysis of specific samples from dead group (**B**), moribund group (**C**), and survived group (**D**) of *Macrobrachium nipponense*.

As an unsupervised pattern recognition method for multidimensional statistical analysis, PCA is often used as a preliminary method to understand the overall metabolic differences between each group and the degree of variation between samples within the group. The PCA of metabolic profiles for hepatopancreas is shown in Figure 1. It can be clearly observed that the metabolites of different survival states under hypoxia were accumulated, whereas the metabolites in the control group were dispersed (Figure 1A). The metabolic patterns of the moribund and dead groups were clearly different from the control group (Figure 1B,C). However, this trend was not shown in the comparison between the survived group and the control group (Figure 1D). 

### 3.2. Significant Metabolites in Response to Hypoxia 

A total of 540 metabolites were detected in three comparisons. The number of significantly different metabolites is listed in Table 1. The different metabolites were compared between the different physiological states and the control group. A total of 108 differential metabolites were found between the control group and the dead group, 76 (70.3%) were down-regulated, and 32 (29.7%) were up-regulated. In the comparison between the control group and the moribund group, there were 86 differential metabolites, in which 61 (70.9%) metabolites were significantly decreased and 25 (29.1%) were significantly increased. Of the 48 differential metabolites between the control group and the survived group, 32 (66.7%) metabolites significantly decreased and 16 (33.3%) metabolites significantly increased. 

**Figure 2:**
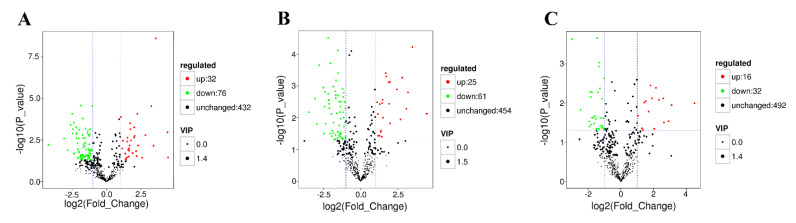
Volcanic map of differential metabolites from three comparisons between control and dead group (**A**), control and moribund group (**B**), and control and group (**C**) of *Macrobrachium nipponense*. The *x*- and *y*-axis are the log2-fold change and the log10 *p*-value of student’s T test between the two compared groups. Red points represent up-regulated metabolites. Green points represent down-regulated metabolites. Black points represent detected but insignificant differences.

According to the fold change (FC) value, the differential metabolites in the three comparisons are shown by volcano plots (Figure 2). Cluster analysis is a multivariate statistical analysis method used to classify samples or indicators that can easily and intuitively observe data characteristics. First, the quantitative results of all samples were logarithmically transformed (log_2_), and then the metabolites with the same or similar expression patterns were clustered. The clustering results are shown in Figure S1.

**Figure 3:**
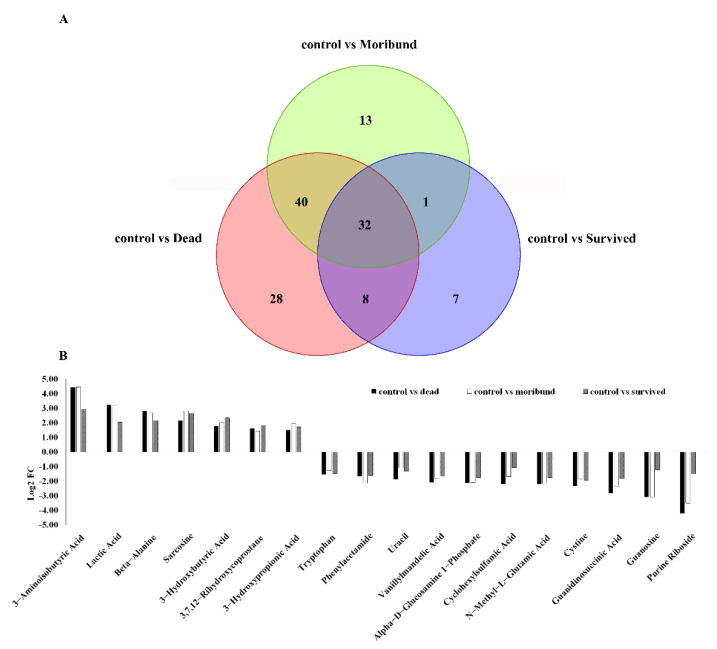
Venn diagram of different metabolites in each group (**A**) and expression of common differential metabolites in three comparisons (**B**) examined in *Macrobrachium nipponense*.

There were 32 common significant metabolites among the three comparisons (*p* < 0.05). After removing unidentifiable metabolites, only 18 common differential metabolites were found. These substances were analyzed according to their Log_2_ FC values (Figure 3B). 

### 3.3. KEGG Pathway Analysis 

**Figure 4:**
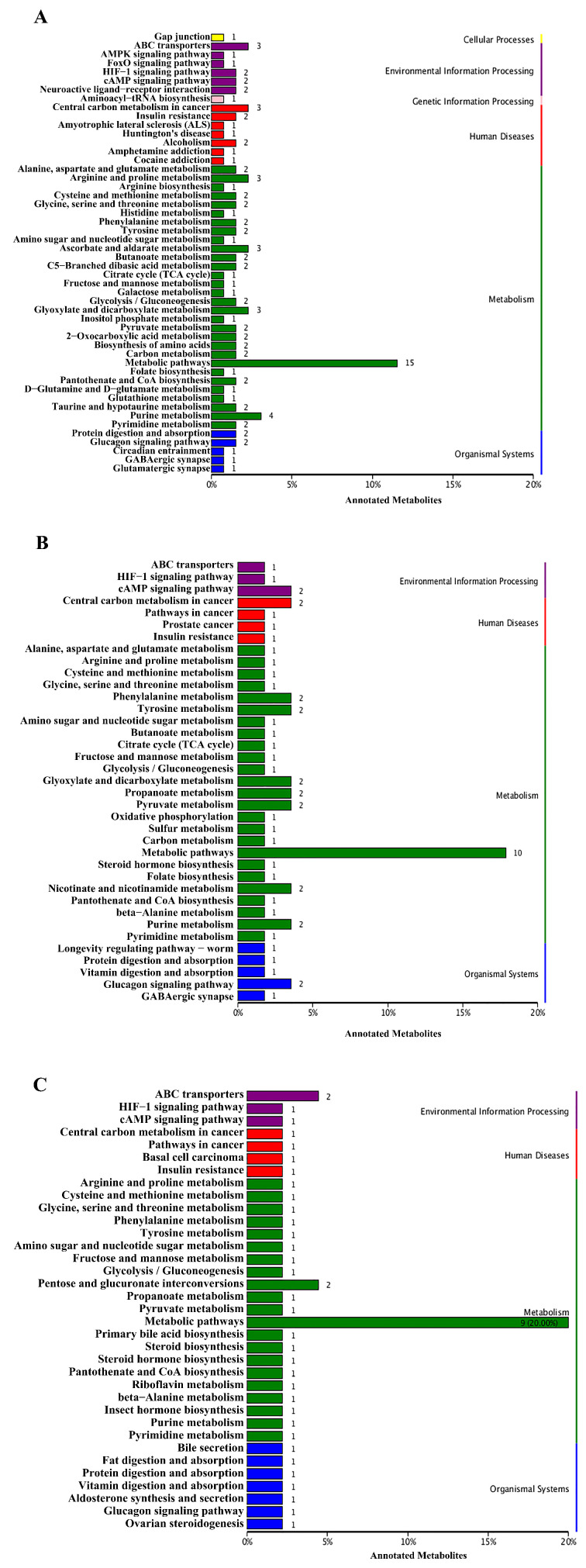
KEGG enrichment of different metabolites between control and dead group (**A**), control and moribund group (**B**), and control and survived group (**C**) in *Macrobrachium nipponense*.

All the different metabolites were enriched in different metabolic pathways through KEGG analysis. Most pathways occurred in the comparison between the control group and the dead group (81 pathways). The other two comparisons (control group vs. moribund group, control group vs. survived group) had 37 and 35 pathways, respectively. In total, six types of KEGG pathways were identified in the comparison between the control group and the dead group by enriching metabolites, including environmental information processing, cellular processes, genetic information processing, metabolism, organismal systems, and human diseases. In addition to cellular processes and genetic information processing, other types of pathways also appeared in the remaining two comparison groups, but there were differences in the number of enrichment (Figure 4).

### 3.4. Gene Expression Validation 

**Figure 5:**
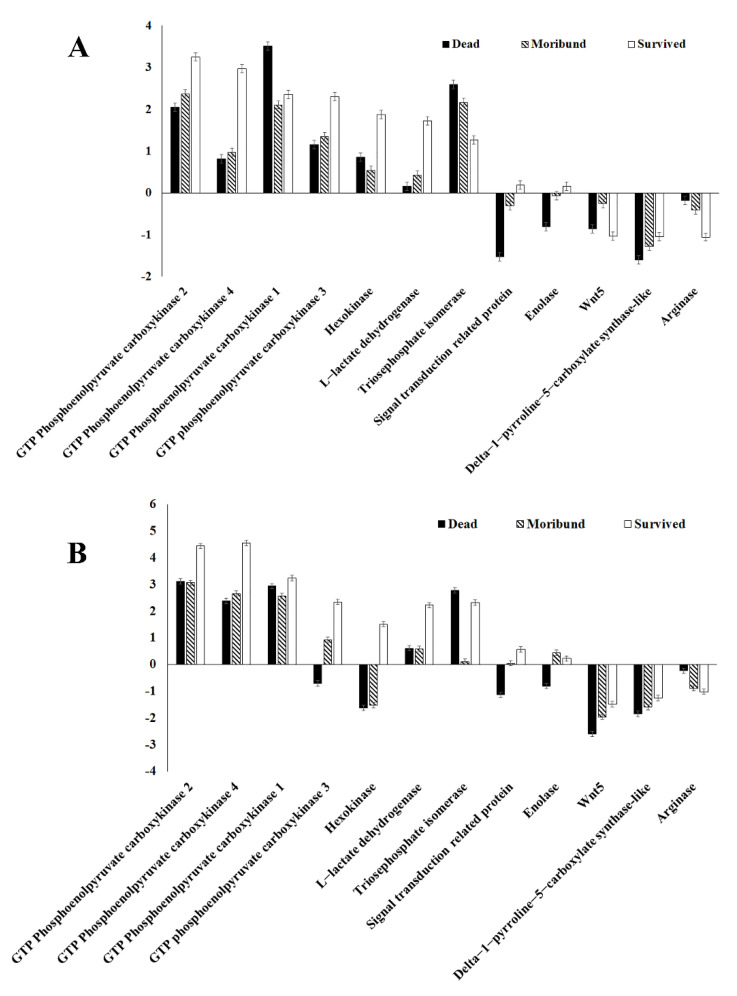
Metabolism validation of selected genes by RNA-seq (**A**) and qRT-PCR (**B**) in *Macrobrachium nipponense*.

According to the result of screening, 18 common differential metabolites were found with relevant annotation. According to the combined analysis with the previous transcriptome database, the metabolites with no related differential gene expression were removed, and the research gradually focused on lactic acid and beta-alanine, which have high differential expression. By retrieving transcriptome data, 12 DEGs were selected to verify the accuracy of the data. The expression trend of these 12 DEGs was consistent with the screened results (Figure 5). 

### 3.5. Subsequent Experimental Verification under Hypoxia 

**Figure 6:**
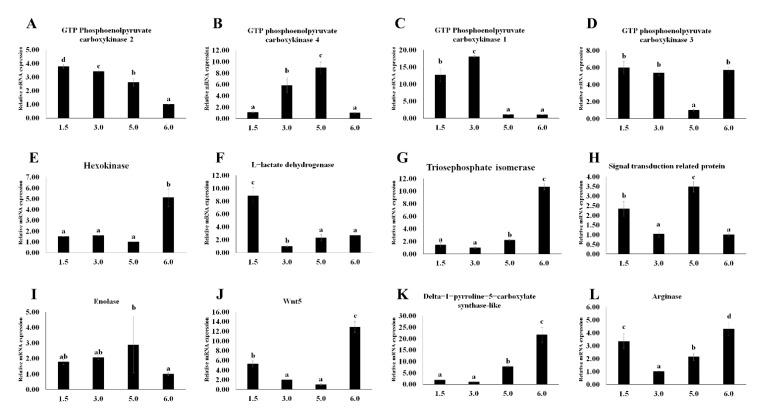
Expression of selected genes in different oxygen concentrations in *Macrobrachium nipponense* by qRT-PCR. (**A**): *Mn-PEPCK2,* (**B**): *Mn-PEPCK4,* (**C**): *Mn-PEPCK1,* (**D**): *Mn-PEPCK3,* (**E**) *Mn-HK,* (**F**) *Mn-LDH,* (**G**) *Mn-TPI*, (**H**) *Mn-STP,* (**I**) *Mn-ENO,* (**J**) *Mn-Wnt5,* (**K**) *Mn-P5CS,* (**L**) *Mn-ARG.* Data are shown as means ± SD of three replicates in various tissues. Statistical analyses are shown with one-way ANOVA. Different letters indicate a significant difference of the same gene in different tissues (*p* < 0.05).

The 12 DEGs mentioned above were also used in subsequent hypoxia experiments. Half of these 12 DEGs showed significant differences between control and other oxygen conditions, including GTP phosphoenolpyruvate carboxykinase-2(*Mn-PEPCK2*), hexokinase (*Mn-HK*), triosephosphate isomerase(*Mn-TPI*), *Mn-Wnt5*, delta-1-pyrroline-5-carboxylate synthase-like(*Mn-P5CS*), and arginase(*Mn-ARG*) (*p* < 0.05, Figure 6A,E,G,J,K,L). The expression of some DEGs at different oxygen concentrations showed no significant difference between the control group and other groups (*p* > 0.05, Figure 6B–D,F,H,I). In these DEGs, the expression profiles of *Mn-HK* and enolase (*Mn-ENO*) confirmed that there is no significant difference in different hypoxic conditions (*p* > 0.05, Figure 6E,I). 

**Figure 7:**
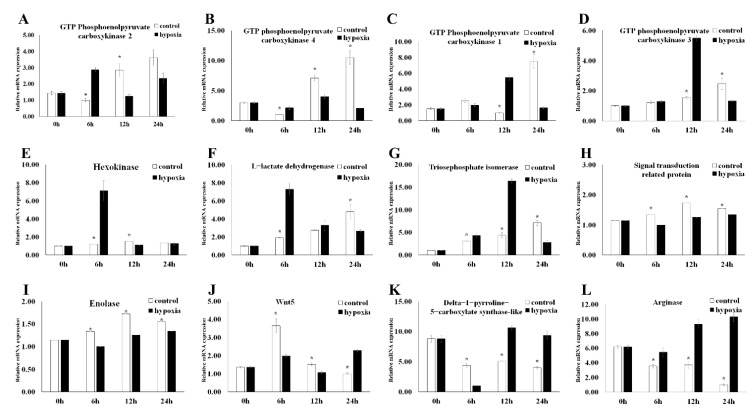
Expression of selected genes in *Macrobrachium nipponense* at different time points under hypoxia by qRT-PCR. (**A**): *Mn-PEPCK2,* (**B**): *Mn-PEPCK4,* (**C**): *Mn-PEPCK1,* (**D**): *Mn-PEPCK3,* (**E**) *Mn-HK,* (**F**) *Mn-LDH,* (**G**) *Mn-TPI*, (**H**) *Mn-STP,* (**I**) *Mn-ENO,* (**J**) *Mn-Wnt5,* (**K**) *Mn-P5CS,* (**L**) *Mn-ARG.* Data are shown as means ± SD of three replicates in various tissues. Statistical analyses are shown with paired *t* test. Data indicated with asterisks are significantly different (*p* < 0.05) between treatment and control groups.

Compared with the control group, six DEGs showed a trend of first increasing and then decreasing, including *Mn-PEPCK1*, *Mn-PEPCK3*, *Mn-PEPCK4*, *Mn-HK*, *Mn-TPI*, and L-lactate dehydrogenase (*Mn-LDH*) (Figure 7B–G). Even within the same gene family, the expression pattern of *Mn-PEPCK2* is different from that of other subtypes. After the decrease of *Mn-PEPCK2* expression, an upward trend showed again in the expression profile (Figure 7A). The same trend was also shown in *Mn-Wnt5* (Figure 7J). However, the expression pattern of the remaining DEGs was completely opposite to that of the DEGs mentioned above, which first decreased and then increased (Figure 7H,I,K,L). 

## 4. Discussion 

The DEMs were divided into two categories according to the level of expression: significantly up-regulated and significantly down-regulated. The top three DEMs were selected for further screening. The DEMs were combined with data from the previous transcriptome libraries, and those were not enriched with DEGs that were removed. Finally, lactic acid and sarcosine were selected as differential metabolites for further experimental analysis. These two metabolites were significantly up-regulated and were highly ranked. Through the joint analysis with the transcriptome, it was found that there were several DEGs in the metabolic pathways of these two metabolites. 

Lactic acid is widely known as the main product of anaerobic metabolism. When oxygen is scarce, pyruvate cannot be further oxidized and is reduced to lactic acid, a process called anaerobic fermentation. Previous studies have identified 12 enzymes in the glycolysis pathway that catalyze the anaerobic fermentation of glycogen into lactic acid [26]. In general, the expression level of lactic acid will increase when the tissue cannot obtain enough oxygen or process oxygen fast enough. This trend has also been confirmed in this experiment. It is clearly shown that lactic acid increased in a hypoxic environment. Meanwhile, lactic acid accumulates with the gradual deterioration of survival status (Figure 3B). As the research has developed in this field, *Mn-LDH* and *Mn-ENO*, which are directly related to lactate, have become more prominent in the field of vision. *Mn-LDH* reduces pyruvate to lactic acid under hypoxia, preventing the inhibition of glycolysis and ATP synthesis due to the buildup of pyruvate [27]. It was clearly observed that *Mn-LDH* decreased with the gradual deterioration of survival status under hypoxia (Figure 5). The same trend was shown in rats [28]. In line with a previous study, *Mn-LDH* is considered in this study to be the A subtype among the many subtypes of *LDH* [28]. Compared with the *LDH* in *Eriocheir sinensis* (H. Milne Edwards, 1853), which increased gradually after hypoxia, *Mn-LDH* in *M. nipponense* decreased six hours after reaching a peak (Figure 7F) [29]. These results suggest that with the deterioration of survival status and the extension of time in hypoxia, large amounts of lactate dehydrogenase are consumed in response to hypoxia. This inference is demonstrated in Figure 7F. When facing a mild and moderate hypoxic environment (3.0 ± 0.2 mg O_2_ L^−1^ and 4.5 ± 0.2 mg O_2_ L^−1^, respectively), *Mn-LDH* in *M. nipponense* can adequately transform the pyruvate accumulation caused by hypoxia. However, *Mn-LDH* in *M. nipponense* needs to increase its secretion to reduce the increased pyruvate severe hypoxia (1.5 ± 0.2 mg O_2_ L^−1^). This also explain why pyruvate does not appear in the common DEMs of the three groups. Pyruvate was only found in the comparison between the dead group and control groups.

As one of the many downstream genes of the HIF-signaling pathways, *ENO* increases the tolerance of hypoxia by participating in anaerobic metabolism [30,31]. It was clearly observed that the expression of *Mn-ENO* significantly increased with the decrease of oxygen concentration (Figure 6, *p* < 0.05), indicating that *ENO* plays an important role in promoting the anaerobic metabolism in *M. nipponense* after hypoxia. The rising trend of *Mn-ENO* in *M. nipponense* is the same as it is in *Ruditapes philippinarum* (Adams et Reeve, 1850) [27]. However, different from *R. philippinarum*, the expression of *Mn-ENO* in the hypoxia group was always lower than that in the control group (Figure 7, *p* < 0.05). Combining the results above, it can be reasonably inferred that when challenged with a hypoxic environment, abundant *Mn-ENO* in *M. nipponense* will be preferentially consumed to increase anaerobic metabolism to adapt to the hypoxia. This theory has also been mentioned in human cancer studies [32]. A gene with hypoxia response elements (HRE) that is recognized by HIF as *ENO* has been, *HK* has also been studied widely [33]. Although the relationship between *HK* and *HIF* has been researched in previous studies by RNA inference technology [34], there is no research about the specific expression profile of *HK* in *M. nipponense* under hypoxia. It was clearly observed that *Mn-HK* increased after 6 h of hypoxia and then decreased (Figure 7E, *p* < 0.05). This trend also appeared in other crustaceans under different environmental conditions, such as *Carcinus maenas* (Linnaeus, 1758) and *Litopenaeus vannamei* (Boone, 1931) during the moult cycle, and *L. vannamei* under different levels of salinity [35,36,37]. Combined with the result under different hypoxia conditions, it is indicated that *Mn-HK* will be consumed with the duration of hypoxia, and *Mn-HK* is considered to be one of the early genes responding to hypoxia.

As an indispensable enzyme in the gluconeogenic pathway, *PEPCK* has been regarded as essential in humans and mice [38,39]. Interestingly, compared with the previous transcriptome analysis, four *PEPCK* genes were found in the joint analysis of the metabolome and transcriptome [13]. Although the expression patterns of these *Mn-PEPCK*s (except *Mn-PEPCK3*) were different under different hypoxic conditions, the overall trend of the subtypes is that the expression in the hypoxia group is significantly higher than it is in the control group (Figure 6A–D, *p* < 0.05). Previous experiments have shown that hypoxia could induce gluconeogenesis in the liver of rats and humans through the increase of *PEPCK* expression under hypoxia [40]. These experiments further demonstrated that gluconeogenesis is regulated by *PEPCK* [41,42,43]. The same expression patterns also appeared in *M. nipponense*, indicating that *Mn-PEPCK1, Mn-PEPCK2*, and *Mn-PEPCK4* are involved in promoting gluconeogenesis. However, the expression of *Mn-PEPCK3* appeared significant under different time points, indicating that *Mn-PEPCK3* is also participating in the adaptation to hypoxia. Therefore, it is reasonable to hypothesize that different subtypes of genes may be activated in response to hypoxia under different conditions. Alternatively, *PEPCK* may deal with hypoxia by processes other than gluconeogenesis. Recent research has obtained two subtypes of *PEPCK* in *L. vannamei*, which showed completely opposite expression profiles in response to hypoxia [44]. These results indicate that *Mn-PEPCK3* might be similar to one of these two *PEPCK* genes and that there are more than two subtypes of *Mn-PEPCK* in *M. nipponense*. It should be noted that *PEPCK* is the only DEG that ranked highly both in transcriptome analysis alone and when combined with metabolome analysis. These results suggest that *PEPCK* may play an essential role in *M. nipponense* in their response to hypoxia.

As an important enzyme involved in gluconeogenic, *TPI* is studied widely. *TPI* can catalyze the conversion between dihydroxyacetone phosphate and glyceraldehyde-3-phos-phate (G3P) [45]. Previous studies have demonstrated that *TPI* is an ideal internal standard gene for RT-PCR in *Fenneropenaeus chinensis* (Osbeck, 1765) under challenges by *White Spot Syndrome Virus* (WSSV) [46]. However, *TPI* was very unstable when *Penaeus vannamei* (Boone, 1931) responded to high temperature [47]. These results indicate that the stability of *TPI* has significant differences in different populations. It is clearly shown that *Mn-TPI* increased with the length of hypoxia and increased with the content of oxygen in *M. nipponense*. This indicates that, compared with *TPI* in *F. chinensis*, *Mn-TPI* is more similar to *TPI* in *P. vannamei*. In previous studies, it was found that glycolysis-gluconeogenesis-related enzymes, including triosephosphate isomerase, G3P, *LDH*, acetyl-coenzyme A (acetyl-CoA), significantly decreased under light conditions. Relevant experiments have confirmed that the synthesis pathway of glycolysis/gluconeogenesis was inhibited [48]. This opposite trend suggested that the synthesis pathway of glycolysis/gluconeogenesis was induced under hypoxia, and *Mn-TPI* is considered to be used for pyruvate metabolism in response to hypoxia.

Previous studies have suggested that *Wnt5* plays various roles in vertebrate development and participates in initiating meiosis for ovarian follicular growth in mammals [49,50,51,52]. However, in invertebrates, *Wnt5* shows a completely different function. Six *Wnt* genes were obtained in *L. vannamei*, including *LvWnt4*, *LvWnt5*, *LvWnt6*, *LvWnt7*, *LvWnt10*, and *LvWnt16*. It has been proved that *LvWnt*5 was regulated by *WSSV* in *L. vannamei* [53]. In the subsequent study, more *Wnt genes* were found participating in the immune response in *L. vannamei* [54]. As a negative regulator of virus-induced innate immune responses [55], *Wnt5* was found to be related to hypoxia for the first time *in M. nipponense.* Compared with the expression patterns of six *LvWnts*, the expression trend of *Mn-Wnt5* is familiar with *LvWnt5* in response to WSSV and ammonia–N stress [53,56]. This result indicated that *Mn-Wnt5* may be involved in the immune response caused by the accumulation of toxic substances under hypoxia. However, a link between *Wnt5* and hypoxia has been discovered before. A previous study had shown that *Wnt5* increased significantly under ischemia and reperfusion injury in rat liver BRL-3A cells [57]. The same trend was also observed in ischemic brain damage of newborn rats under hypoxia [58].

*P5CS* is a mitochondrial enzyme that catalyzes the coupled phosphorylation and reduction-conversion of L-glutamate to delta-1-pyrroline-5-carboxylate (P5C) [59]. *P5CS* plays an important role in the biosynthesis of proline, ornithine, and arginine [60]. Previous studies have proved that people can develop progressive neurodegeneration due to a deficiency of *P5CS* [60]. The knockout of *P5CS* has indicated that it is necessary for mitochondrial respiratory complex organization [61]. With the development of research, *P5CS* in wheat has been found to respond to changes in salinity in the environment [62]. However, the expression profile of *P5CS* in wheat is the complete opposite of *Mn-P5CS* in *M. nipponense* under hypoxia. This result indicated that *P5CS* may have several subtypes or it may perform different functions in different species. Although there is no research about *P5CS* in crustaceans, there are some studies about P5C dehydrogenase (*P5CDh*) in crustaceans, especially in *L. vannamei* [63]. Unlike *P5CS*, which catalyzes the reduction of glutamic acid (Glu) to glutamic-γ-semialdehyde and pyrroline-5-carboxylate, *P5CDh* converts the latter two to the former [64]. Different from the expression of *P5CDh* in *L. vannamei* in response to environmental changes, the expression of *P5CS* in *M. nipponense* increased gradually after reaching the lowest level 6 h after hypoxia. Previous studies have found that *LvP5CDh* plays a key role in immune defense and antioxidant response [63]. These results suggest that *P5CS* and *P5CDh* may act as a pair of antagonistic genes to regulate biological homeostasis in response to environmental changes, including changes in oxygen, pH, and salinity.

Arginase (*ARG*) is an enzyme that can metabolize L-arginine into L-ornithine and it exists widely in various organisms. L-arginine is the substrate for nitric oxide (NO) synthase that generates NO with L-citrulline [65]. In animals with closed tube circulation, endogenous NO maintains vascular integrity by maintaining vasodilator tone and modulating vascular smooth muscle cell proliferation [66,67,68]. Two subtypes of arginase are reported in humans, *ARG*Ⅰ and *ARG*Ⅱ. *ARG*Ⅰ is a cytosolic enzyme that is highly expressed in the living and *ARG*Ⅱ is a mitochondrial protein [69]. These two subtypes both exist in the lung [70]. It was clearly observed that *ARG*Ⅱ in pulmonary artery smooth muscle cells (PASMC) significantly increased under hypoxia [69]. Subsequent experiments have identified that the silencing of *HIF-2*, but not *HIF-1*, prevents the activation of *ARG*Ⅱ by hypoxia [71]. Although there are various reports about the relationship between hypoxia and *ARG* in humans, few studies are related to the involvement of *ARG* in hypoxia in crustaceans. This manuscript is the first one to propose a relationship between *ARG* and hypoxia in *M. nipponense*. Combined with the result that there are two subtypes of *HIF-1* in *M. nipponense*, the focus of subsequent experiments will be understanding the relationship between the two *HIF-1s* and *ARG*.

## 5. Conclusions

In this manuscript, metabolism and transcriptome analyses were combined for the first time to analyze the different physiological states of *M. nipponense* under hypoxia. Through the screening of common metabolites across three comparisons and in combination with differential genes, 12 genes closely related to hypoxia in *M. nipponense* were detected. In addition to those widely known in hypoxia, GTP phosphoenolpyruvate carboxykinase, hexokinase, L-lactate dehydrogenase, and triosephosphate isomerase were found in this manuscript. Genes, such as Mn-Wnt5, delta-1-pyrroline-5-carboxylate synthase-like, enolase, and arginase have not previously been proved to be related to hypoxia in crustaceans, or previously found in *M. nipponense*. This manuscript fills a gap in hypoxia transcriptome analysis and reversely deduces genes related to hypoxia from metabolites. It enriches our understanding of the hypoxia response model of *M. nipponense* and provides a theoretical basis for the subsequent resolution of apoptosis caused by hypoxia.

## Figures and Tables

**Table 1:** Number of significantly different metabolites down-regulated (Down) and up-regulated (Up) in different comparisons examined in *Macrobrachium nipponense*.

	All Different Metabolites	Down	Up	Number of Pathways
Control vs. Dead	108	76	32	81
Control vs. Moribund	86	61	25	37
Control vs. Survived	48	32	16	35

## Data Availability

Data is contained within the article and supplementary materials.

## Supplementary Materials

The following supporting information can be downloaded at: https://www.mdpi.com/article/10.3390/antiox11010036/s1. Figure S1: Cluster analysis of three comparisons between control and dead group (A), control and moribund group (B), and control and survived group (C) in *Macrobrachium nipponense*, Table S1: Primers for qRT-PCR.
