# Supplementary material for: Integrated Metabolomics and Transcriptomic Analysis of Hepatopancreas in Different Living Status Macrobrachium nipponense in Response to Hypoxia
Source: Antioxidants (Basel). 2021 Dec 24;11(1):36. doi: 10.3390/antiox11010036 (PMC8772856; doi:10.3390/antiox11010036)
Supplement: Supplementary file 1 [file antioxidants-11-00036-s001.zip › antioxidants-1499311-supplementary.pdf]

Table S1. Primers for qRT-PCR.

Target mRNA	Sequence (5′-3′)
Enolase -F	TGATCTCACCAGTTCTAAGACCG
Enolase -R	CGGTATCTGAAGCAATTCAAGCA
Hexokinase -F	TGGAACAAATGCTTGCTACATGG
Hexokinase -R	AGCTCTTCTTGAAACGCATTCAC
Triosephosphate Isomerase -F	CCCTTCCTGCTACCTGACTTATG
Triosephosphate Isomerase -R	CAAAAATGGTCCTCCTTTCTGGG
GTP Phosphoenolpyruvate Carboxykinase 1-F	TAGTGTATTGGGGCAATGGTTCA
GTP Phosphoenolpyruvate Carboxykinase 1-R	GACCAAAGGTACGACAGTTTGTG
GTP Phosphoenolpyruvate Carboxykinase 2-F	TTAACATCATGCAGAAGGCTGGA
GTP Phosphoenolpyruvate Carboxykinase 2-R	AGATCATCAGGTGACATCCACTT
L-Lactate Dehydrogenase -F	CTCGTTTCAGATTCCACTTGTCC
L-Lactate Dehydrogenase -R	TTGTGCATCTCGTTGTACTTGTC
GTP Phosphoenolpyruvate Carboxykinase 3-F	GAGACACCAACTGGACTAAGGAG
GTP Phosphoenolpyruvate Carboxykinase 3-R	CCGAAGTTGTAACCGAAGAATGG
GTP Phosphoenolpyruvate Carboxykinase 4-F	AAGAACCTGGATAAACGTGTGGA
GTP Phosphoenolpyruvate Carboxykinase 4-R	CCTATACATCAGGGTTGCAGTGA
Signal Transduction-Related Protein -F	AAACGACAGATAAGCTACCGGAT
Signal Transduction-Related Protein -R	ACATGGAACGTGAAGAGAAGTGA
Arginase -F	GGAGCGAATAAAACGCTGGAAAT
Arginase -R	GAAGCAATTATGTGTCGCTCGTT
Delta-1-Pyrroline-5-Carboxylate Synthase-Like -F	GAAGATGAAGAGACAGCAGACCA
Delta-1-Pyrroline-5-Carboxylate Synthase-Like -R	AAAATCAGACGCTACTCCACAGT
Wnt5-F	TCATGTGAAAAGGGCTACAGACA
Wnt5-R	GAAGGTGAAGTCTGCTCTTCTGA

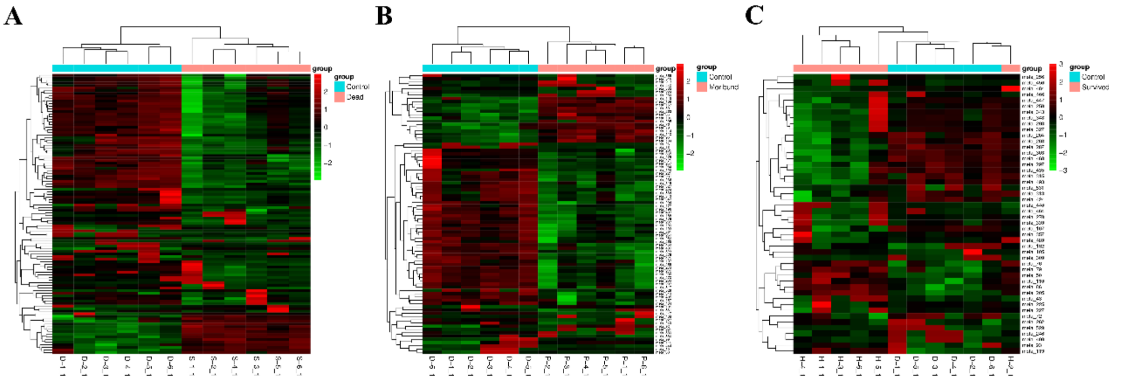

Figure S1. Cluster analysis of three comparisons between control and dead group (**A**), control and moribund group (**B**) and control and survived group (C).
